# Classification of Cracks in Composite Structures Subjected to Low-Velocity Impact Using Distribution-Based Segmentation and Wavelet Analysis of X-ray Tomograms

**DOI:** 10.3390/s21248342

**Published:** 2021-12-14

**Authors:** Angelika Wronkowicz-Katunin, Andrzej Katunin, Marko Nagode, Jernej Klemenc

**Affiliations:** 1 Department of Fundamentals of Machinery Design, Faculty of Mechanical Engineering, Silesian University of Technology, Konarskiego 18A, 44-100 Gliwice, Poland; angelika.wronkowicz-katunin@polsl.pl; 2 Faculty of Mechanical Engineering, University of Ljubljana, Aškerčeva 6, SI-1000 Ljubljana, Slovenia; marko.nagode@fs.uni-lj.si (M.N.); jernej.klemenc@fs.uni-lj.si (J.K.)

**Keywords:** barely visible impact damage, damage classification, real oriented dual-tree wavelet transform, X-ray computed tomography

## Abstract

The problem of characterizing the structural residual life is one of the most challenging issues of the damage tolerance concept currently applied in modern aviation. Considering the complexity of the internal architecture of composite structures widely applied for aircraft components nowadays, as well as the additional complexity related to the appearance of barely visible impact damage, prediction of the structural residual life is a demanding task. In this paper, the authors proposed a method based on detection of structural damage after low-velocity impact loading and its classification with respect to types of acting stress on constituents of composite structures using the developed processing algorithm based on segmentation of 3D X-ray computed tomograms using the **rebmix** package, real-oriented dual-tree wavelet transform and supporting image processing procedures. The presented algorithm allowed for accurate distinguishing of defined types of damage from X-ray computed tomograms with strong robustness to noise and measurement artifacts. The processing was performed on experimental data obtained from X-ray computed tomography of a composite structure with barely visible impact damage, which allowed better understanding of fracture mechanisms in such conditions. The gained knowledge will allow for a more accurate simulation of structural damage in composite structures, which will provide higher accuracy in predicting structural residual life.

## 1. Introduction

Carbon fiber reinforced polymer (CFRP) composites, which are increasingly wide-spread in numerous industrial branches, found their primary applications in aircraft and aerospace vehicles due to their excellent strength-to-mass ratio, resistance to corrosion and numerous chemical agents, flexible forming and manufacturing capabilities, etc. Despite their numerous advantages, one of the main problems of these materials during operation is their susceptibility to impact loading (see e.g., [1,2,3]). The most dangerous type of impact damage from an operational point of view is the so-called barely visible impact damage (BVID), which appears at low-velocity impact (LVI) loading and results in extended internal damage that is often not recognizable on a surface. This creates a necessity of periodic inspections of elements with such a type of damage using advanced non-destructive testing (NDT) techniques, which enable identification of internal damage as well as monitoring of possible propagation of such damage.

Damage caused by LVI loading, although often not visible on a surface of a CFRP structure, especially when this surface is covered by paint or other type of coatings, causes an extended net of various types of cracks and delaminations. Due to the complex stress state, different types of cracks appear during such a loading, including vertical, diagonal, and horizontal (known as delaminations). An analysis of acting stresses and a characterization of BVID in terms of fracture mechanics can be found in [4]. Cracks and delaminations resulting from LVI can cause significant reductions in numerous properties of the material, mainly compressive strength and resistance to buckling [5,6], which are the key parameters of composite structures in aviation.

Since BVID remains a serious problem for the operation of composite elements and structures in numerous industrial branches, a variety of NDT techniques were adjusted to detect this type of damage. Taking into account the practical reasons, the detection and localization of BVID is performed primarily using ultrasonic testing (UT), infrared thermography (IRT), and guided waves (GWs) [7,8,9,10,11,12,13,14,15,16], less often acoustic emission (AE), shearography, and other NDT technqiues [8,17,18,19]. The mentioned techniques are characterized by various sensitivity to BVID, resolution of the obtained results, and other parameters, but are usually selected for inspection because of their simplicity of application. Nevertheless, none of the above-mentioned techniques are capable of providing NDT results in a three-dimensional (3D) representation and with sufficient resolution to investigate the nature and fracture mechanisms occurring during LVI, which is necessary to investigate damage mechanisms in an impacted structure. This investigation is a necessary step in the reconstruction of BVID currently developed by the authors of this study to predict the residual life of composite structures after LVI [4,14,20].

Fortunately, X-ray computed tomography (XCT) provides necessary properties of the inspection results, including representation of the resulting tomograms in a form of 3D grayscale arrays with high resolution, which makes it possible to identify all types of cracks mentioned above. Despite the high cost of testing, XCT found a wide applicability in problems related to the detection, localization and identification of structural damage in composites [6]. However, due to the complexity of an internal architecture of composite structures, presence of scanning artifacts, and difficulties with a proper interpretation of the obtained results, tomograms are usually subjected to further post-processing. An overview of problems that appear during XCT measurements and processing of the resulting tomograms can be found in [21]. Within post-processing methods, one can mention those based on image segmentation techniques, which reveal very good results in the cases where cracks are remarkably distinguishable in resulting tomograms and a texture of internal architecture of a composite, is not dominating. Such segmentation can be performed even using built-in tools in a software dedicated to viewing and initial post-processing of results, which was demonstrated in [3,22,23,24,25]. However, in many cases, the resulting tomograms contain visible reinforcement characteristics and generally low contrast compared to a polymeric matrix [21], which makes it difficult to segment damage from them (see [6], for example). This requires the development of advanced image processing algorithms to extract necessary diagnostic information.

To date, different algorithms for the classification of structural damage from NDT tests have been proposed. A simple binary segmentation approach to cracks identification problem from tomograms was proposed by Léonard et al. [26]. A more advanced cracks identification algorithm based on the semi-automatic ’seed-growth’ approach, was proposed by [27], where the analyzed cracks were clearly distinguishable on a background of tomograms. An advanced algorithm of segmentation of tomograms was proposed by Yu et al. [28], where they successfully classified the constituents of a composite and various types of debonding. Another classification algorithm proposed by Mardanshahi et al. [29] was based on artificial neural networks. In this study, the authors successfully identified matrix cracks in composite structures. Several other advanced algorithms have recently been developed to detect and classify cracks [30,31,32] from noisy NDT results.

During the previous studies performed by the group of the authors from the Silesian University of Technology, several algorithms for extraction of diagnostic information from tomograms were developed. In [33], the authors developed the damage classification algorithm, which was based on image segmentation, filtering, and morphological operations, and was able to distinguish cracks and delaminations from tomograms of damaged composite structures. Later, this approach was enhanced in terms of image segmentation and the texture/background removal procedures were added. The developed algorithm was applied to CFRP structures with BVID [4,14]. However, based on the above literature survey, two major emerging problems with XCT tomograms damage classification can be defined. The first problem is related to the proper interpretation of NDT results, which is ultimately performed by a human. This problem is connected with a second one, namely, in the current times we usually deal with large amounts of data. Manual analysis is a difficult, responsible, and time-consuming task, thus, the automation of interpretation is a desired tool in numerous branches. In numerous cases, such as during the analysis of aircraft inspection results, a human cannot be substituted by an algorithm due to the appropriate protocols and responsibility [34]. However, the development of an effective and automated algorithm, which is able to provide a valid interpretation of NDT results in a short time period, is a desired supporting tool for inspectors. In addition to the acceleration of processing and interpretation of such results, such algorithms have an additional advantage—the decrease of the number of errors made by a human in the interpretation step, which is confirmed in some studies [35]. In the times of the fourth industrial revolution, it is essential to develop effective and automated tools for the interpretation of the results, which, in addition to the already mentioned benefits, will allow for a significant reduction in inspection costs. The bottleneck of this general problem is the need for the development of effective, fast, and smart solutions to automate the process of interpretation of inspection results, which is the fundamental supporting tool for high-level NDT inspectors.

The following study is an attempt to solve the above-defined problems with the interpretation and automation of the processing of XCT testing results. The specific aim of this study was to develop an effective and fast image processing algorithm capable of retrieving diagnostic information on damage from tomograms and classifying the damage recovered according to the types of stresses that act during LVI of a composite structure. To achieve the defined goal, a two-step processing and classification algorithm was developed, which is based on color-based segmentation implemented in the Rough-Enhanced-Bayesian MIXture estimation (REBMIX) algorithm and the real oriented dual-tree wavelet transform used for identification of the directions of crack propagation in the analyzed composite structure with BVID and further classification of these cracks with respect to acting stresses. This made it possible to better understand the character of damage after LVI in composites as well as to automatize the decision process on types of cracks, which makes it possible to significantly improve a speed of classification and to eliminate human mistakes during this process.

## 2. Specimen and Testing Methods

### 2.1. Specimen Preparation and Introducing Impact Damage

The CFRP specimen considered in this study was manufactured and supplied by the Sexcraft s.c. (Helenów, Poland). The matrix of the specimen was made of LG700 epoxy resin by GRM Systems s.r.o. (Olomouc, Czech Republic). The plain weave Toray T700 carbon fabric with the weight of 160 g/cm${}^{2}$ was manufactured by the Toray Composite Materials America, Inc. (Tacoma, WA, USA) and used in the composite as the reinforcement. A composite sheet was manufactured using VARTM resin infusion technology with 10 layers of the reinforcement oriented in the same direction (0° with respect to the global coordinate system). The material properties obtained in the previously performed experimental studies for this material can be found in [20]. The specific dimensions of the considered specimen were as follows: 100 × 100 × 2.5 mm, which corresponds to planar dimensions and thickness.

BVID was introduced into the specimen using the in-house drop weight testing machine, which allowed performing automatic adjustment of test parameters based on the input of the desired impact energy value. According to the results of previous studies [14,20], the case of impact energy of 20 J with the hemispherical impactor with the diameter of 10 mm was selected as representative and met the criteria of a bare visibility of an impact damage [36]. The view of the testing facility and the impacted specimen is presented in [4].

### 2.2. X-ray Computed Tomography Tests

**Figure 1:**
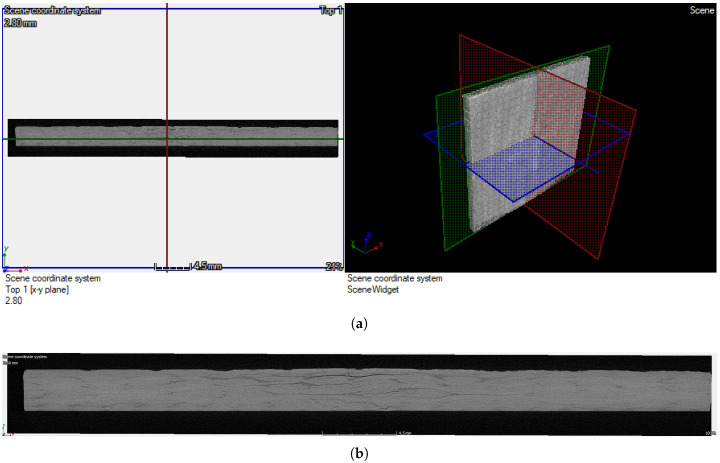
The view of the XCT scan of the tested specimen (**a**), and the exemplary slice in the normal direction to the specimen’s surface (**b**).

The scanning using the XCT technique was performed by Machinefish Materials & Technologies Sp. z.o.o. Sp. k. (Wrocław, Poland). Before scanning, the specimen was cut to dimensions of 40 × 70 × 2.5 mm based on the results of the ultrasonic testing [4] in such a way that the cutting process had no influence on the introduced BVID. The scanning was performed using the XT H 225 ST industrial computed tomography scanning system (Nikon, Tokyo, Japan) with the 225 kV reflection and 180 kV transmission targets. The scanning volume was defined as 40 × 40 × 2.5 mm, which covered all elements of the resulting damage that appeared during LVI and resulted in dimensions of a single voxel of 0.022 × 0.022 × 0.01 mm${}^{3}$. The resulting scan was obtained in a form of a grayscale 3D array, which was sliced in a direction normal to the surface of the dedicated software specimen using the myVGL 2.2 (Volume Graphics GmbH, Heidelberg, Germany) and then exported in a form of a sequence of 2D images. A view of the XCT scan with indicated slicing planes is presented in Figure 1a, while the exemplary slice is presented in Figure 1b.

The above-presented slices indicate the presence of various types of damage, including delamination, matrix, and interface cracks. No other types of damage were observed, such as reinforcement damage or perforation damage, since the load during impact testing was comparatively low. However, the types of the observed damage are characterized by variable visibility and thus detectability, which implies the necessity of their proper identification and classification.

## 3. Cracks Recognition

In crack recognition problems, one of the key issues is the image segmentation [37,38]. High quality image segmentation is important as it can significantly simplify further object detection and recognition [39,40]. Within the current problem, the objects of interest are cracks and voids, which need to be separated from the background as efficiently as possible. The processing procedures were applied to the 2D slices of a 3D grayscale array representing the damaged structure (see Section 2.2 for more details). This means that *x*, *y*, and *z* pixel coordinates are at the disposal, together with the corresponding gray colors, as integers in the range from 0 to 255.

For image segmentation, the clustering capabilities of the **rebmix** package are evaluated in the paper. From the tomographic 3D arrays, only information on pixel gray colors is intentionally used in order to simplify and speed up clustering to the highest possible level.

### 3.1. Short Overview on rebmix Package

The main capabilities of the **rebmix** R package are the finite mixture estimation, clustering, and classification. R is a programming language aimed at solving different problems in statistics, machine learning, data mining, etc. The **rebmix** package has been freely available at https://CRAN.R-project.org/package=rebmix, where one can get a reference manual and vignette with examples, source code, and others. It is based on the Rough-Enhanced-Bayesian MIXture estimation (REBMIX) algorithm [41,42,43,44]. The version used in this study is 2.13.1 from 28 July 2021.

**Figure 2:**
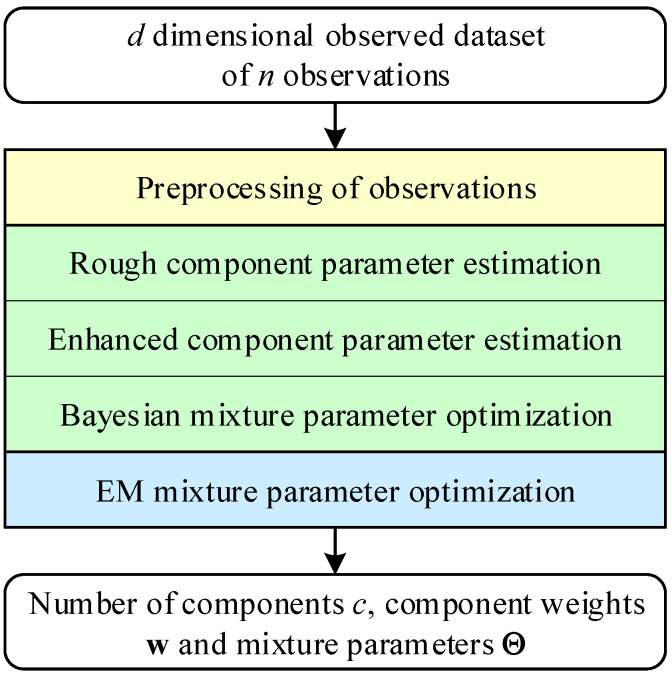
Flowchart of REBMIX & EM.

The first step in image segmentation is a finite mixture estimation, as shown in Figure 2. Let $y_{1},\ldots ,y_{n}$ be an observed *d* dimensional dataset of size *n* of continuous or discrete vector observations $y_{j}$. Each observation is assumed to follow the predictive mixture density

(1) $$f\left(y|c,w,\Theta \right)=\sum_{l=1}^{c}w_{l}f\left(y|\theta_{l}\right)$$

with conditionally independent component densities

(2) $$f\left(y|\theta_{l}\right)=\prod_{i=1}^{d}f\left(y_{i}|\theta_{il}\right)$$

indexed by the parameter vector $\theta_{l}={\left(\theta_{1l},\ldots ,\theta_{dl}\right)}^{⊤}$. The objective is to obtain the number of components *c*, component weights $w_{l}$ summing to 1 and component parameters $\theta_{l}$ in such a way that they represent the global optimal solution.

The task is far from trivial and is achieved iteratively by applying the numerical procedure thoroughly described in [41,45,46,47]. Besides REBMIX there exist other prominent algorithms for finite mixture estimation. The Expectation Maximization (EM) algorithm introduced in [48] is one of them. Today, after more than 40 years, it is still one of the most popular algorithms for statistical pattern recognition. It has turned out to be favorable to combine both algorithms [42]. The REBMIX provides *c*, w, and Θ, which tend to be close to optimal, while the EM algorithm aims to reach the global optimal solution. This way, the best is gained from the algorithms.

### 3.2. Image Segmentation by rebmix Package

For image segmentation, we assume d=1 and y=y, where *y* represents the value of gray color of the pixel. Equation (1) thus yields

(3) $$f\left(y|c,w,\Theta \right)=\sum_{l=1}^{c}w_{l}f\left(y|\theta_{l}\right)$$

Furthermore, the data set $y_{1},\ldots ,y_{n}$ of size n=width×height represents gray colors as integers, where width and height determine image size in pixels. This means that any image can be preprocessed into a histogram containing no more than v=256 nonempty bins. Therefore, the histogram can be understood as an unbiased and optimized substitute for the data set. The preprocessing of observations for image segmentation in Figure 2 should therefore always be set to "histogram", ymax to 255.5, ymin to −0.5, and K representing the number of bins *v* to 256, which yields unbiased binning of observations in the **rebmix** package at bin width of size

$$\begin{array}{c} h=\frac{y_{\max}-y_{\min}}{v}=1. \end{array}$$

Pilot testing has further pointed out that the Gumbel component probability density

$$f\left(y|\theta_{l}\right)=\frac{1}{\sigma_{l}}\exp\left\{-\frac{y-\mu_{l}}{\sigma_{l}}-\exp\left\{-\frac{y-\mu_{l}}{\sigma_{l}}\right\}\right\}$$

can provide better image segmentation as normal or Weibull parametric families. The 3D tomographic arrays have been processed to estimate the finite mixture parameters in Equation (1) and to cluster the images.

The 3D tomographic arrays have been processed to estimate the finite mixture parameters in Equation (1) and to cluster the images by calling the methods REBMIX and RCLRMIX consecutively. The maximum number of components has been set to 16, the information criterion to "BIC" and the EM strategy to "exhaustive". See help("REBMIX") and help("RCLRMIX") in **rebmix** for details. Such a numerical setup has resulted in clusters representing cracks and voids only, background only, and sometimes also cracks and voids partially contaminated by the noise from the background. It has not yet been possible to completely restore barely visible cracks if the gray colors of the cracks blend with the background. This topic has been left over to be dealt with in the future. The clusters containing cracks and voids contaminated by background noise could here serve as a good starting point for some improvements.

**Figure 3:**
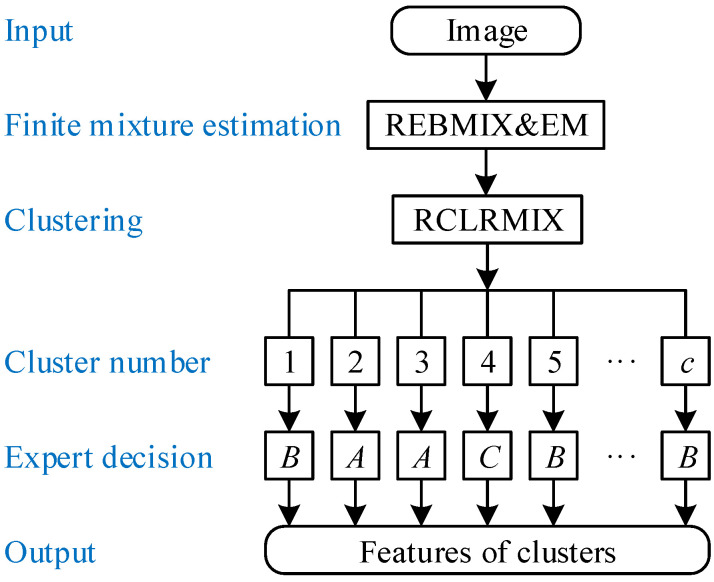
Training of image segmentation.

The training of image segmentation is shown in Figure 3. It requires input, e.g., a 3D tomographic array consisting of a sequence of z=1,…,depth images (layers). Each image Dataset enters the training individually and is processed in two steps. In the first step, it is converted to the predictive mixture density f(y|c,w,Θ) by calling the REBMIX method

rng = range(Dataset)

EM <- new("EM.Control", strategy = "exhaustive", K = rng[2] - rng[1])

rebmixest <- REBMIX(Dataset = Dataset, Preprocessing = "histogram",

cmax = 16, Criterion = "BIC", pdf = "Gumbel", K = 256,

ymin = -0.5, ymax = 255.5, EMcontrol = EM)

The method range calculates the minimum and maximum gray color values in the Dataset. The method new creates an object EM of type "EM.Control". The maximum number of components has been set to 16, the information criterion to "BIC", and the EM strategy to "exhaustive". See help("REBMIX") in **rebmix** for details.

In the second step, the image is clustered. The number of clusters is always less or equal than the number of components. The cluster numbers s=1,…,c are assigned to the coordinates of the pixels *x* and *y* by the RCLRMIX method

rebmixclr <- RCLRMIX(x = rebmixest)

The clustering is based on the Bayes decision rule. The RCLRMIX method returns an object rebmixclr of type "RCLRMIX" containing information on the clustered image. See help("RCLRMIX-class") for further reading. In this particular case no merging of clusters has been used, although it is available in **rebmix**. The cluster structure is thus affected solely by the information criterion BIC, the maximum number of components $c_{\max}=16$, and the parametric family type "Gumbel" in the REBMIX method. Such a numerical setup has resulted in clusters representing cracks and voids only, background only, and sometimes also cracks and voids partially contaminated by the noise from the background. It has not yet been possible to completely restore barely visible cracks if the gray colors of the cracks blend with the background. This topic has been left over to be dealt with in the future. The clusters containing cracks and voids contaminated by background noise could here serve as a good starting point for some improvements.

Next, an expert opinion on image labeling is requested. The cluster can be labeled as cracks and voids only *A*, background only *B*, or cracks and voids contaminated by the noise *C*. Finally, an output is formed for each image. It is composed of the features to follow: image number *z* and vector of length *c* containing cluster numbers 1,…,c, labels designations *A*, *B*, or *C* of individual clusters together with minimum, median, and maximum gray color values encountered in the part of the image associated to cluster *s*. Training can be performed on all images, or a carefully selected set of images, or by a randomly selected set of images.

Once training is complete, the minimum and maximum gray color values of the clusters designated as *A* are carefully analyzed. If the gray colors of the cracks and voids differ from the background to some extent, image segmentation can be automated. In this respect, color filters are proposed. We can have, for example, three filters [0,29] representing almost black voids, [37,78] denoting dark gray cracks, and [217,255] standing for almost white cracks. This means that the pixel with coordinates *x*, *y*, and *z* of color value within [0,29] or [37,78] or [217,255] is designated as a crack or void. Otherwise, it means background. The automation of image segmentation is then trivial. The filters have so far been identified by an expert. In the future, filter definition is aimed to be automated, at least to some extent.

### 3.3. Analysis of the Crack Recognition Results

The results obtained from the recognized anomalies in 2D CT slices were used to produce a 3D array, which corresponds to the entire composite specimen.

**Figure 4:**
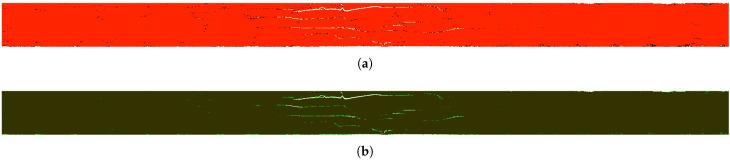
The selected 2D slice with labeled clusters (**a**), and the same slice after preclassification (**b**).

Figure 4a presents the selected 2D slice with all labeled clusters, while Figure 4b shows the same slice with regions preclassified as damage based on the experts’ knowledge.

**Figure 5:**
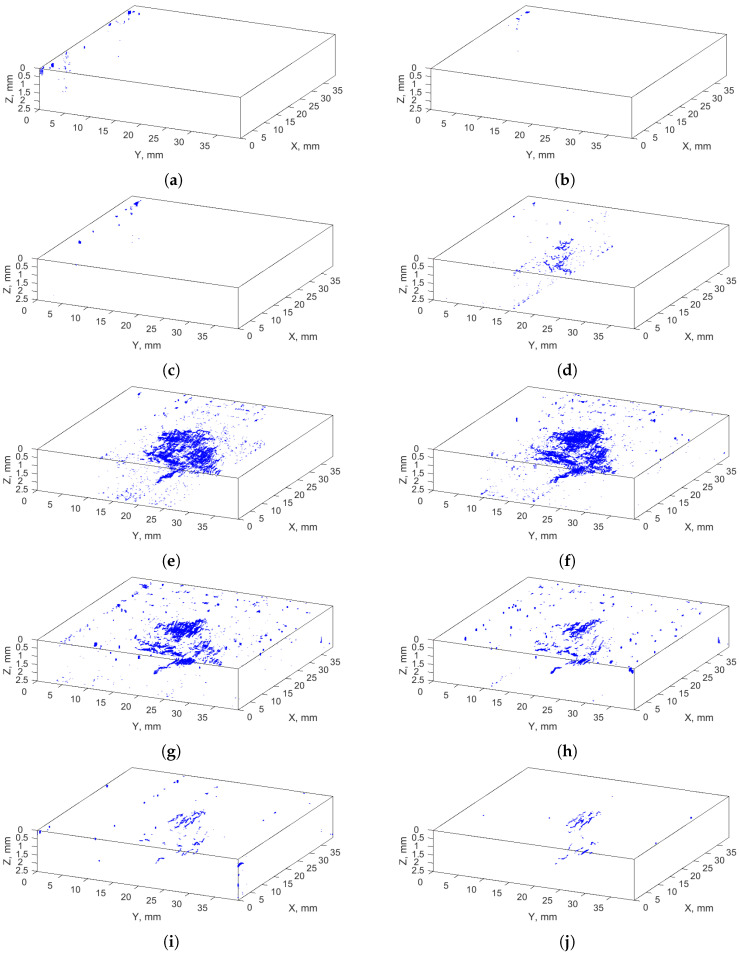
Recognized anomalies—clusters with positive labels nos.: (**a**) 2; (**b**) 4; (**c**) 5; (**d**) 6; (**e**) 7; (**f**) 8; (**g**) 9; (**h**) 10; (**i**) 11; (**j**) 12.

**Figure 6:**
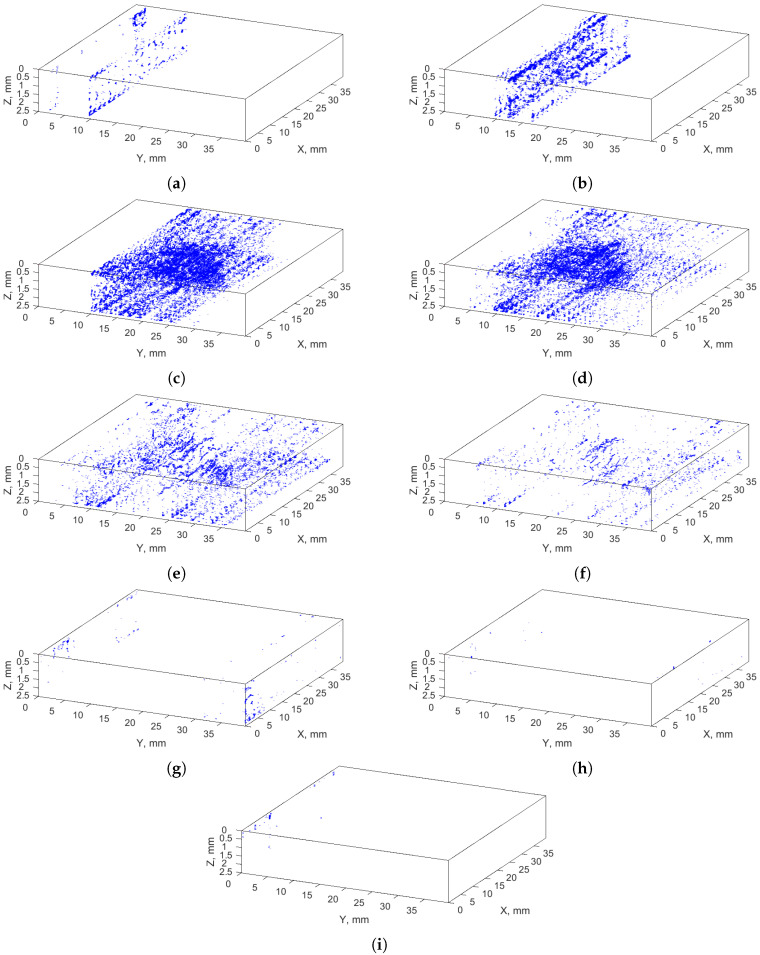
Recognized anomalies—clusters with negative labels nos.: (**a**) −4; (**b**) −5; (**c**) −6; (**d**) −7; (**e**) −8; (**f**) −9; (**g**) −10; (**h**) −11; (**i**) −12.

Figure 5 and Figure 6 present 3D views of the recognized anomalies divided into individual clusters with positive and negative labels, respectively. The analysis of the recognized areas indicated that cracks are included in the clusters: 6–12, whereas clusters 2, 4, 5, and −10, −11, −12 contain air voids (identified as shapes close to spherical), and clusters −4–−9 mostly represent noise (rounding the cracks and areas of healthy composite fibers with similar pixels’ values). Representation of the resulting clusters in the form of 3D arrays makes it possible to observe entire set of a given type of features present in an XCT scan, which makes it possible to distinguish anomalies from identified cracks.

**Figure 7:**
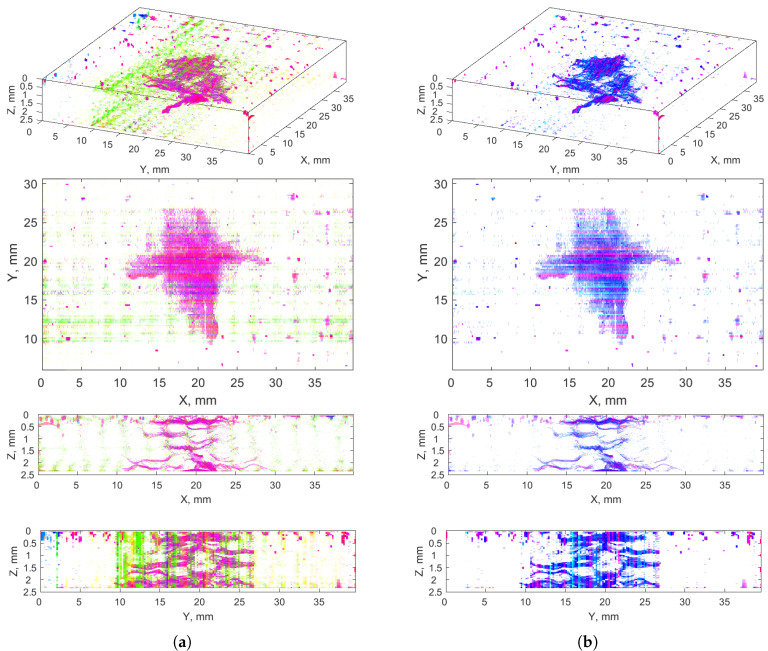
Recognized anomalies: (**a**) all clusters (nos. −4–12); (**b**) selected clusters with cracks (nos. 6–12).

Figure 7 presents 3D and side views of the cumulative clusters of the anomalies (the clusters are marked with different colors), namely all the clusters (**a**) and the clusters selected as cracks (**b**). A comparative analysis of these visualizations proved the correctness of the selection of the crack clusters, since an excess of noise is removed while the crack regions remained in the resulted images. Some pixels representing the external surroundings of the cracks (areolas) could be omitted due to excluding the clusters −4–−9, however, the level of their noise was too high to include them to the result.

The analysis of Figure 7b also indicated that the crack classification into vertical, horizontal, and diagonal ones is not possible based on the obtained results of the crack recognition determined from the pixels’ values (no relationship is noticeable between the cluster numbers and the crack directionality); thus, additional image processing operations are needed.

## 4. Cracks Classification

The classification of types of identified cracks by acting stresses in a composite structure during impact loading is generally based on the estimation of the directionality of these cracks, which correspond to various mechanical interactions during such a loading. Based on the results of the previous studies [4], three groups of directions were assumed:

- vertically oriented cracks, being the result of indentation and bending stresses on the opposite side with respect to an impacted surface;
- skew cracks with an inclination of ca. 45° with respect to an impacted surface, which is a result of the acting of shear stresses during impact loading;
- horizontally oriented cracks, that is, delaminations, are the result of the action of shear stresses on the existing vertically oriented and skew cracks in a matrix of a composite structure, triggered by exceeding the critical stress values at the fiber/matrix interfaces of a composite.

Having defined three possible orientations in the tested composite structure, it is essential to select a tool which allows distinguishing cracks of these orientations. This step of the algorithm was based on the 2D real oriented dual-tree wavelet transform (DTWT), which reveals appropriate directional selectivity together with the low noise level during decomposition (see the analysis presented in [49]).

The obtained results of the crack recognition were filtered using three binary masks, defined based on the detail coefficients obtained after a decomposition using DTWT with areas possibly containing damage of various directionality. This operation was divided into two stages: extraction of the masks based on the raw (gray-scale) data of CT slices; and then filtering the recognized cracks in the previous stage using the masks.

**Figure 8:**
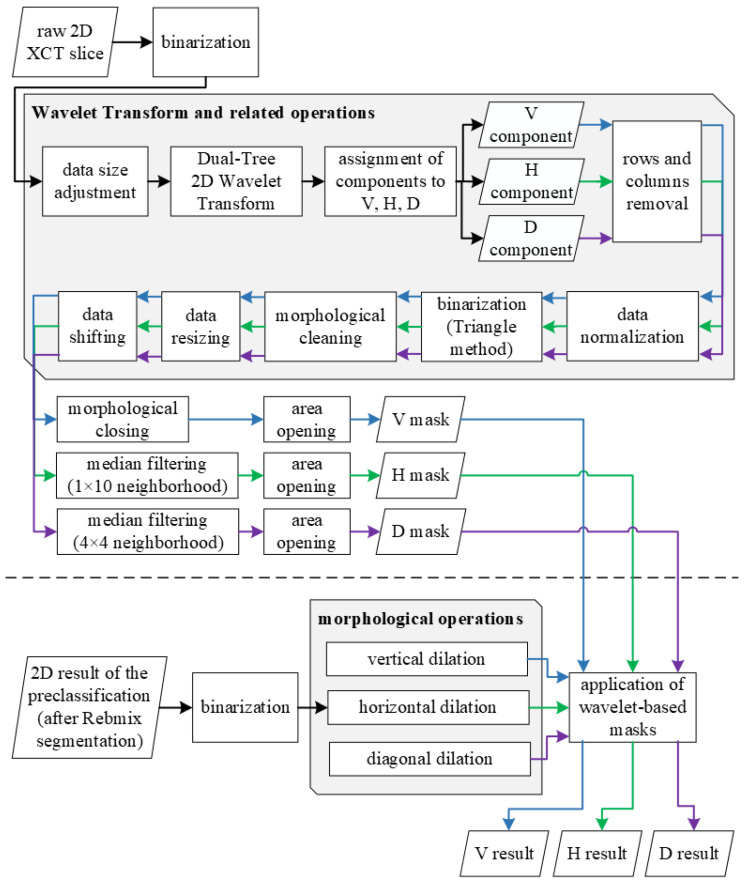
The algorithm of cracks classification.

A scheme of the algorithm steps is illustrated in Figure 8, and the description is presented in Section 4.1 and Section 4.2, respectively, for the two stages of the algorithm.

### 4.1. Wavelet-Based Masks

**Figure 9:**
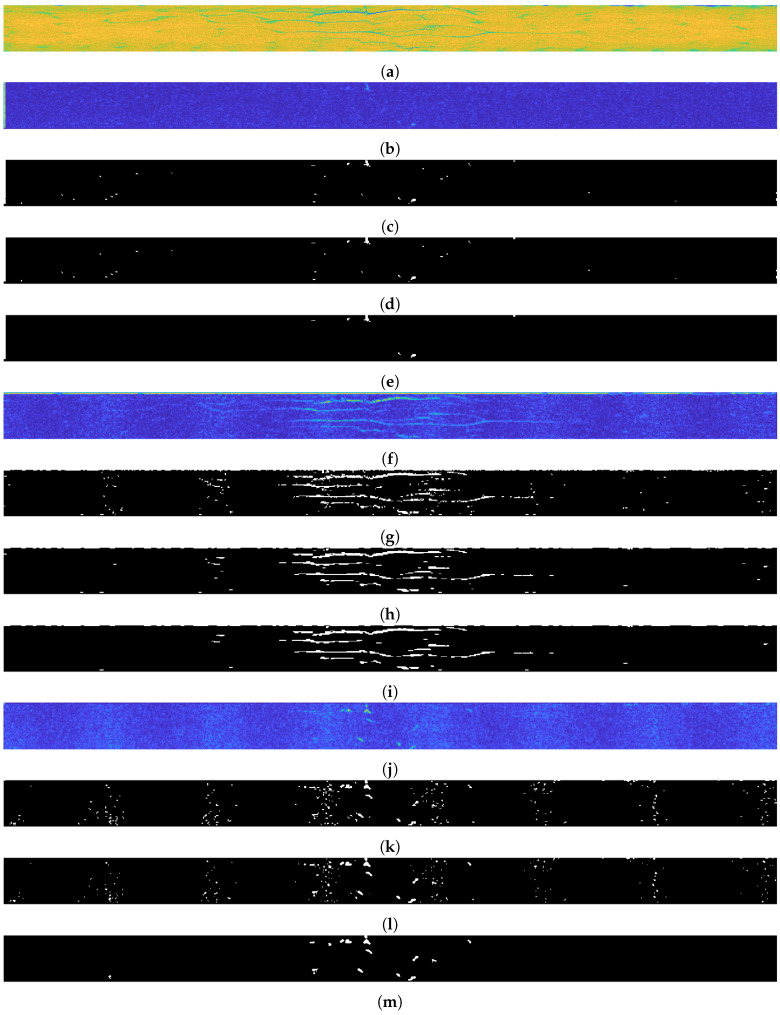
The original slice (**a**) and the results of WT and post-processing—slice 84: (**b**–**e**) vertical; (**f**–**i**) horizontal; (**j**–**m**) diagonal.

The mask extraction example is presented on a selected 2D XCT slice (see Figure 9a) no. 113 from 198). Raw 2D XCT slices were initially subjected to a binarization in such a way that the threshold was defined at zero, which means that all nonzero pixel values were assigned to unities. Next, the adjustment of a 2D XCT slice size was performed according to the requirements of the applied DTWT algorithm, since DTWT is of a dyadic type, which means that the dimensions of the input data should be a multiplicity of a power of 2. For this purpose, empty rows and columns were added to the binarized 2D XCT slices, resulting in their dimensions of 128 × 2048 pixels.

After initial processing, each slice of the sequence considered of the 2D XCT slices was subjected to further processing using the DTWT algorithm. DTWT was selected in this study due to its high directional selectivity, as well as its robustness to noise [50], which was confirmed in previous studies related to structural damage identification [49]. A single-level decomposition using the 2D real oriented dual-tree wavelet transform using 10-tap Kingsbury Q-shift filters resulted in 8 sets of directional detail coefficients. The selection of a single-level decomposition was forced by the need to retain appropriate spatial resolution of the resulting data after decomposition, while the selection of a wavelet basis represented by the above-mentioned filters was based on previous analyzes of performance of various directional wavelet transforms and filterbanks cited above. The resulting wavelet coefficients can be grouped into three predominant directions: horizontal *H*, vertical *V*, and diagonal *D*. According to this, the resulting sets of detail coefficients were added up following the directions of sensitivity:

(4) $$\begin{array}{c} d_{H}=d_{3}+d_{6}, \end{array}$$

(5) $$\begin{array}{c} d_{V}=d_{1}+d_{2}, \end{array}$$

(6) $$\begin{array}{c} d_{D}=d_{4}+d_{5}+d_{7}+d_{8}, \end{array}$$

where the numbers in the lower indices denote the subsequent numbers of the absolute values of the detail coefficients *d* obtained after decomposition using DTWT. Next, the resulting values $d_{H}$, $d_{V}$, and $d_{D}$ were assigned to the initial masks. The previously added empty rows and columns used to fulfill the requirements of DTWT were removed (see the result in Figure 9b,f,j) and the resulting data were normalized to 8-bit integers, which was necessary for a further binarization procedure. Binarization was performed using the Triangle method [51], which was empirically selected and revealed the best thresholding results (see Figure 9c,g,k). In the next steps, morphological cleaning was performed in order to filter out single pixels. Then, the original dimensions (reduced due to the application of DTWT) were restored, and a shifting operation was applied in order to eliminate the boundary effect resulting from the application of DTWT. Further operations were focused on additional improvement of the masks in order to enhance the extraction of the vertical, horizontal, and diagonal objects, and remove noise as follows:

- V: morphological closing (dilation and erosion) using a vertically oriented `line’ structuring element (Figure 9d); and morphological area opening in order to remove very small objects (Figure 9e),
- H: median filtering using horizontally oriented filter (Figure 9h); and morphological area opening (Figure 9i),
- D: median filtering using a square shape filter (Figure 9l); and morphological area opening (Figure 9m).

In the end, the resulting 2D masks for the particular 2D XCT slices were stored in a 3D array.

**Figure 10:**
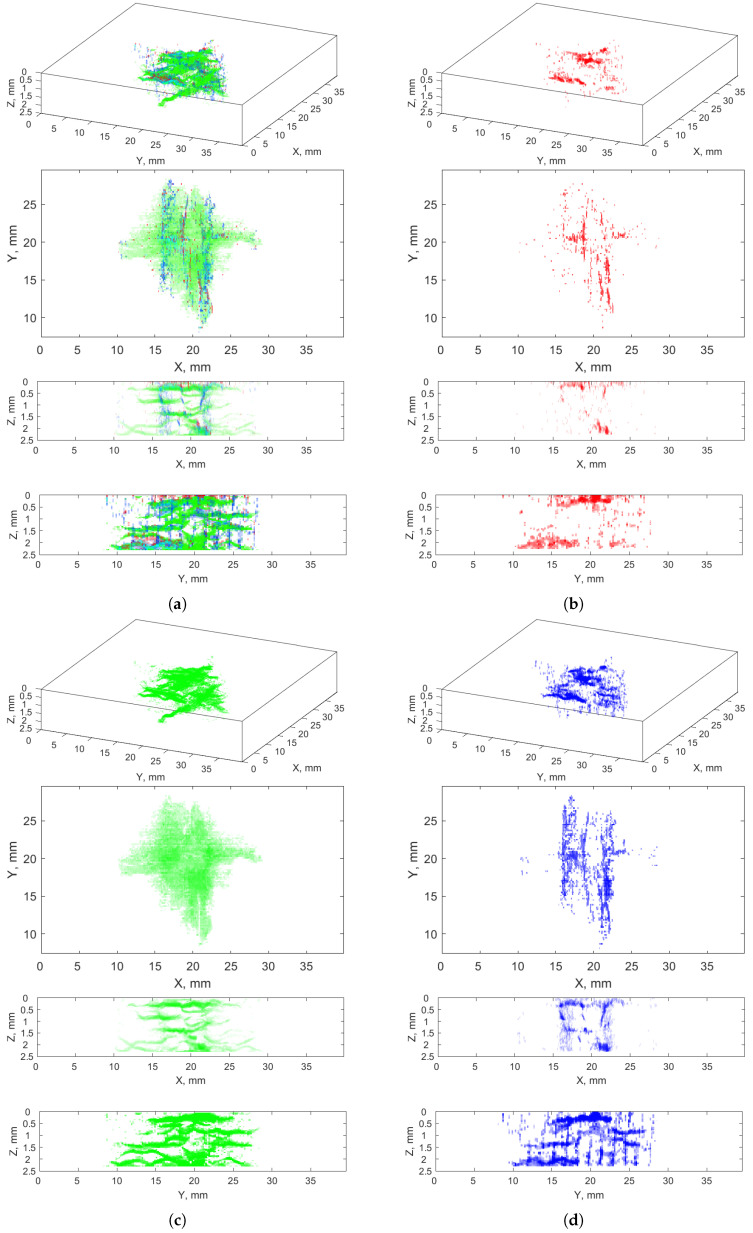
Wavelet-based masks obtained from the implementation of the proposed algorithm for V—vertical, H—horizontal, D—diagonal components: (**a**) V, H, and D; (**b**) V; (**c**) H; (**d**) D.

Figure 10 illustrates the V, H and D masks after building 3D arrays from the obtained 2D masks and, for better readability of the results, removing the background (for this purpose, an additional mask with a 2D shape of damage from the top view was extracted).

### 4.2. Results on Classification of Cracks

**Figure 11:**
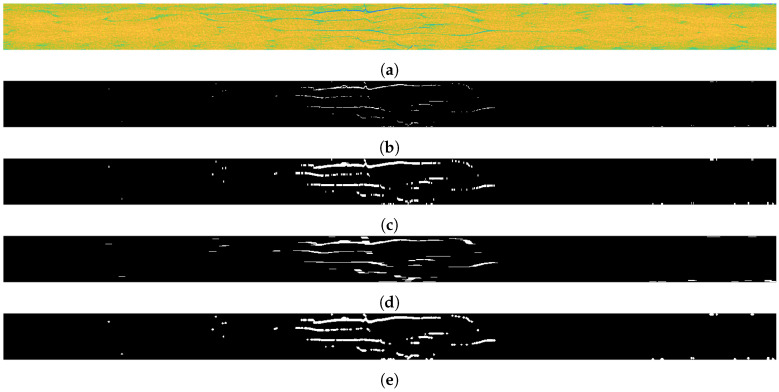
Processing of the results: (**a**) original CT slice; (**b**) recognized cracks; and directional expansion in the direction: (**c**) vertical; (**d**) horizontal; (**e**) diagonal.

Taking into account that some pixels representing cracks could have been omitted during the crack recognition stage after REBMIX&EM segmentation (the clusters with crack surroundings were excluded due to the high level of noise), the recognized cracks (Figure 11b) were binarized (nonzero pixels assigned to ones) and then slightly expanded using a morphological dilation) before filtering with the prepared masks to improve the ability to classify cracks. The image with the recognized cracks was dilated in three ways (using a structuring element oriented vertically (Figure 11c), horizontally (Figure 11d), and diagonally (Figure 11e)), which allowed obtaining three resulted images to be filtered by the corresponding masks. Filtering using the masks was performed by finding common regions between the pairs of images.

**Figure 12:**
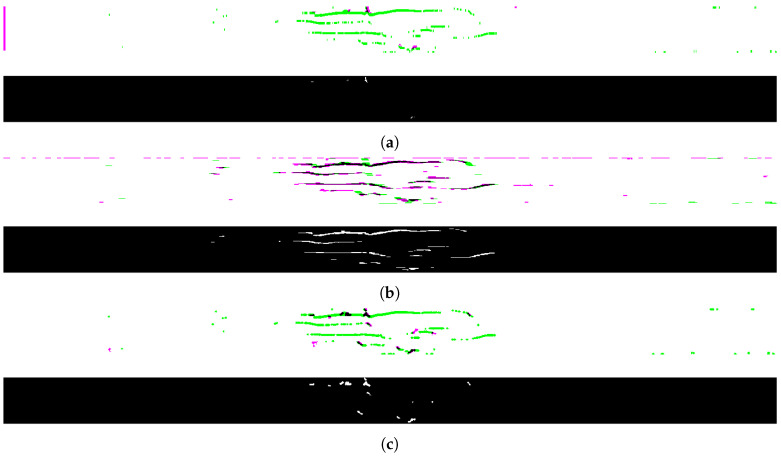
Recognized cracks after masking using: (**a**) V mask: (**b**) H mask; (**c**) D mask.

Figure 12 presents the final 2D results of the exemplary CT slice, i.e., the pixels with components V, H, and D, representing the three classes of cracks considered. The upper images show the differences between the recognized and dilated cracks with the corresponding masks (black regions in the composite image show where the two images have the same intensities; magenta and green regions show where the intensities are different), whereas the lower images present binary results (common regions).

One can observe that the proposed method based on wavelet-based masks ensures flexibility in attribution of cracks to particular classes with respect to their orientation, i.e., delamination (horizontal cracks) visible in Figure 12 contains characteristic disturbances from a linearity, which is caused by the waviness of reinforcement in the tested composites, and thus a delamination follows the fiber/matrix interface. Despite a slight change of angle with respect to the surface of impact, delamination was correctly attributed to the class of horizontal cracks.

An additional problem with the analyzed data is the presence of cases, where different types of cracks are connected to each other. This connection is a natural physical process resulting from the damage mechanics. For example, delamination usually appears by exceeding the critical adhesion stresses between fiber and matrix of a composite and propagates on the interface, but its origin is usually a vertical or a skew crack. Such a situation is visible in Figure 11 for the first delamination from the top. It is noticeable that the origin of this delamination is a vertical crack in the middle of this delamination, which most probably appeared during the indentation of an impactor. Distinguishing merged cracks is a difficult task, considering classical segmentation tools; however, the proposed method allowed attributing different types of cracks to particular classes, which additionally justifies the application of the wavelet-based masks.

**Figure 13:**
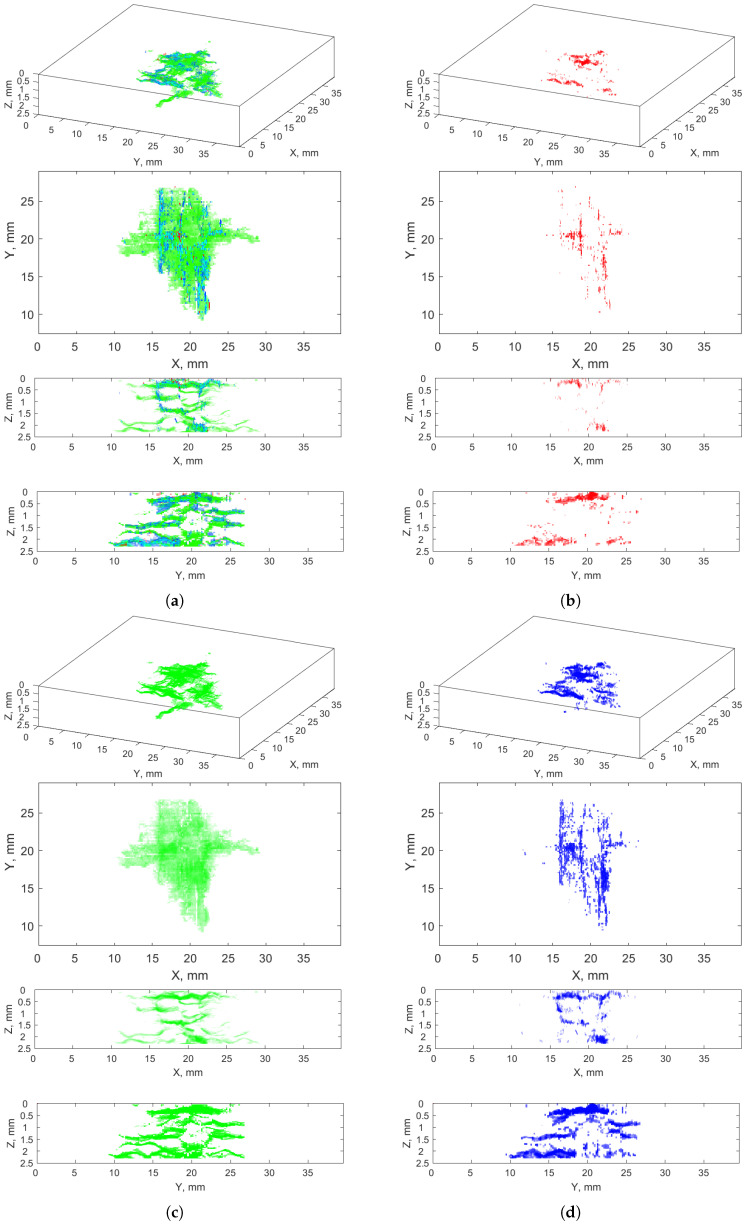
The result of classification of types of cracks resulting from LVI using the proposed method: (**a**) all types of cracks considered in the study, and (**b**) vertical, (**c**) horizontal, (**d**) diagonal cracks.

The final result of a crack classification in the composite structure considered using the proposed method is presented in Figure 13a, where the processed 2D XCT slices were restored to a 3D array. For convenience, particular components V, H, and D attributed to the particular classes of cracks were presented in Figure 13b–d, respectively. Comparing the obtained shape of the BVID with the shape of the reconstructed BVID using another approach of the same structure presented in [4], one can conclude about their high similarity. This means that supporting operations such as morphological operations and filtering itself do not significantly alter the relevant diagnostic information by using wavelet-based masks. However, improving classification remains an open question in this class of problems.

The classification performed allows us to evaluate the nature of a fracture mechanism of the investigated LVI. Vertical cracks (Figure 13b) are observed in the top and bottom regions along the thickness of the analyzed structure, which coincides well with the theoretical considerations. The vertical cracks that appeared in the top region are the result of indentation, while the cracks of this type that appeared in the bottom region are the result of propagation of the shock wave resulting from impact through the structure and the initiation of bending stresses in the bottom layers of a composite. Furthermore, it can be observed that the most vertical cracks appeared on a contour of the impact damage (see, especially, the X-Y view in Figure 13b), which confirms the character of a propagation in the form of a truncated cone typical for this type of damage (see [4,52] for instance).

A class with skew cracks presented in Figure 13d has a similar physical nature, i.e. it is a type of crack that appeared directly due to the impact loading. One can observe a high concentration of these cracks at the indentation location, and this type of crack is dominant in this region. This is due to a combination of transverse and shear stresses acting in the vicinity of the indentation, which resulted in the appearance of cracks with an inclination of ca. 45°. This type of crack is present in the deeper regions of the considered structure, as can be observed in the X-Z and Y-Z views in Figure 13d, however, its character of appearance is transient, i.e., comparing these views with the similar views for delamination presented Figure 13c, one can observe that often skew cracks link particular delaminated areas. This confirms the triggering nature of skew cracks for the appearance of delamination, and they propagate usually from interface to interface.

The last class of cracks—delamination presented in Figure 13c—is observable on the entire thickness of the composite structure considered. This is mainly due to the lowest resistance of a composite to crack propagation on interfaces, which is also the reason for the most extended cracked areas compared to previously discussed types of cracks. As it can be noticed, this type of cracks is of a secondary type, since it is not resulting directly from impact loading, but initiated from the previously appeared cracks of two previously discussed types.

## 5. Conclusions

In the following study, the new method for the detection, identification, and classification of crack types was proposed with respect to their appearance character from XCT scans. The presented example identifies numerous challenges of this task, namely identification of cracks on geometrically complex and variable backgrounds, merging of types of cracks, etc. A combination of the **rebmix** package used at the identification stage of the algorithm as the powerful image segmentation tool as well as wavelet-based masks based on the real oriented dual-tree wavelet transform with the enhanced directional sensitivity allowed for solving this problem successfully, with a simultaneous robustness to noise and measurement artifacts.

However, the proposed method contains several limitations identified during its testing on XCT data. As was observed, it is difficult to detect cracks with the same color intensity as healthy regions, which can be resolved by adding new segmentation criteria, which will be based not only on color intensity, but also on geometric properties. Moreover, the identification of very short cracks, such as vertical cracks in the case considered, remains a challenging task. These limitations and a complete automation of segmentation procedures are challenges that need to be solved in future research activities.

The presented method of identifying and classifying cracks from XCT scans allows us to deeply characterize the mechanistic nature of damage, including a fracture mechanism, which may be considered as a powerful tool for the interpretation of the results of the tests during and after NDT inspections, especially in the aviation ground maintenance. The proposed method is universal and, with insignificant changes, can be applied in numerous other problems, both in enhancement of interpretation of NDT results and other problems and tasks related to image processing, and in particular, retrieving information from complex backgrounds and with complex structure of objects to be identified and classified.

## Data Availability

The raw/processed data are available from the corresponding author upon request. The **rebmix** software is available on https://cran.r-project.org/web/packages/rebmix/index.html, accessed date 13 December 2021.
